# The association between intrinsic breast cancer subtypes, mammography screening and prognosis: a large population-based real world cohort study

**DOI:** 10.1016/j.breast.2025.104507

**Published:** 2025-05-23

**Authors:** Santeri Palmi, Teemu J Murtola, Mika Murto, Heini Huhtala, Otso Arponen, Arja Jukkola

**Affiliations:** a Department of Oncology, Tays Cancer Center, Tampere University Hospital, Tampere, Finland; b Faculty of Medicine and Health Technology, Tampere University, Tampere, Finland; c Department of General Surgery, Waikato Hospital, Hamilton, New Zealand; d Faculty of Social Sciences, Tampere University, Tampere, Finland; e Institute of Clinical Medicine, University of Eastern Finland, Kuopio, Finland

**Keywords:** Breast cancer, Survival, Prognostic factors, Cancer screening, Mammography

## Abstract

**Introduction:**

Breast cancer (BC) as a heterogeneous disease is routinely managed according to its intrinsic subtypes. Mammographic BC screening reduces overall mortality in females. Our aim was to analyze the association between different intrinsic subtypes and mammography screening coverage (pre-screening-aged, screening-aged vs. post-screening-aged), attendance (attendance vs. non-attendance), and means of detection (screen-detected vs. interval BC) and BC survival.

**Materials and methods:**

We used a subpopulation of a registry including all patients diagnosed with invasive BC in Finland between 1995 and 2013. We collected screening results, information on biological characteristics and survival from national registries.

**Results:**

We included 7389 patients with early-stage BC. Compared to luminal A-like subtype, patients with triple-negative BC had the highest risks of death (HR: 1.81, 95 % CI: 1.52–2.15) and BC-related death (HR: 3.16, 95 % CI: 2.43–4.10). The majority of triple-negative BCs were diagnosed after the screening age. HER2-positive (non-luminal) tumors were most likely interval tumors, while the rest of the subtypes were most likely screen-detected. The risk of death was higher in patients with interval cancers compared to screen-detected cases (HR 1.40, 95 % CI: 1.18–1.68) and even higher among patients not attending screening (HR 2.17, 95 % CI: 1.75–2.68); this association was also detected in major subtypes.

**Conclusion:**

In this real-world dataset, triple-negative tumors had the highest risk of death and majority of these tumors were found after the screening age. In screening-aged females, patients with screen-detected tumors had the best survival, while patients with interval tumors and patients not attending screening had the worst prognosis.

## Highlights

• Screen-detected tumors have the best survival.
• Interval tumors and screening non-attendance is associated with worse survival.
• Screening coverage affects survival at least in the most common subtypes.

## 1 Introduction

Globally, breast cancer (BC) is the most frequently diagnosed cancer and the leading cause of cancer-related deaths in females [1]. The prognosis of BC patients has increased dramatically during the last decades due to improved therapies and mammographic screening programs [2].

BC is biologically a heterogeneous disease, and tumors are routinely managed according to their biological factors as recommended by the European Society for Medical Oncology (ESMO) clinical practice guidelines [3]. Tumor subtypes can be defined most accurately using molecular and genetic technologies; specific gene expression profiling methods to determine the subtype are already in clinical practice as a risk stratification tool for a proportion of early-stage BCs [[4], [5], [6], [7]]. Nevertheless, surrogate classification of BC into intrinsic subtypes (luminal A-like, luminal B-like (HER2-negative), luminal B-like (HER2-positive), HER2-positive (non-luminal) and triple-negative) based on immunohistochemical/in situ hybridization methods for the evaluation of hormone receptors (estrogen and progesterone receptors (ER, PR)), human epidermal growth factor 2 (HER2), and proliferation factor Ki-67 is a commonplace practice [3,[8], [9], [10]] providing not only prognostic but also predictive information [9,[11], [12], [13], [14]]. Luminal A-like and luminal B-like (HER2-negative) subtypes together constitute the majority of all cases, whereas slightly over 10 % of tumors are triple-negative. Luminal B-like (HER2-positive) and HER2-positive (non-luminal) tumors account for 5–10 % of tumors each [10,12,13,15]. However, the proportions of different subtypes in early breast cancer are not well defined, and the methodology of classification vary significantly across previous studies [12,13,15]. Luminal A-like tumors have significantly better prognosis regarding disease recurrence and survival than the other subtypes, while triple-negative tumors have the worst prognosis [9,[11], [12], [13]]. The prognosis of both luminal B-like subtypes and HER2-positive (non-luminal) carcinomas is relatively similar up to 5 years after the diagnosis. Yet, in longer follow-up period, the prognosis of luminal B-like tumors worsens in comparison to HER2-positive (non-luminal) tumors reflecting the proportion of late-recurrences and better HER2-targeted therapies in the modern era [[11], [12], [13]].

The goal of mammographic BC screening is to detect tumors earlier and thus reduce mortality. It is estimated that the provision of mammographic screening reduces BC mortality approximately by 20 % in average risk screening-aged females [[16], [17], [18]]. Most of the earlier studies have included screening with both screen-film mammography (SFM) and full-field digital mammography (FFDM), which is the modern imaging approach currently in use [[16], [17], [18]]. The most robust data on mortality reduction with mammographic screening is established in females aged between 50 and 70 years with biennial screening, but there is also proven benefit of screening starting at 40 years of age as well as screening executed annually or triennially [16,17]. BCs detected between the screening rounds are called interval BC and have worse prognosis than screen-detected cancers [19,20]. The incidences of different intrinsic subtypes among screen-detected and interval cancers are not well established. However, previous studies have stablished that HER2-positive and triple-negative subtypes are over-represented in interval cancers [19,20].

Our aim was to assess the association between different intrinsic subtypes, mammography screening coverage (pre-screening-aged, screening-aged vs. post-screening-aged), attendance (attendance vs. non-attendance), means of detection (screen-detected vs. interval breast cancers) and survival.

## 2 Materials and methods

### 2.1 Patients

This work is based on a subpopulation of a retrospective population-based cohort including all females in Finland diagnosed with invasive BC between January 1st, 1995, and December 31st, 2013. Patients with BC were identified and their national identity numbers were collected from a nationwide cancer registry, The Finnish Cancer Registry. Patients with BC were identified with diagnosis codes between C50.1 and C50.9 according to the tenth version of the International Statistical Classification of Diseases and Related Health Problems (ICD-10) manual. Individual social security numbers of patients diagnosed with BC were used to link information from different registries before anonymizing the data.

Data regarding the age at diagnosis, disease extent (localized, locally advanced or metastatic) as well as the patient's participation in screening were collected from the Finnish Cancer Registry and the Finnish Mass Screening Registry. Finnish residents are invited to participate in free-of-charge biennial mammographic screening covering all females aged 50–69 years with a comprehensive participation-rate [2]. The participation to screening has remained high, despite decreasing from 87 % to 81 % during years 1992–2020 [2]. As our goal was to evaluate the survival of early BC patients, the cohort was limited to patients with localized or locally advanced diseases.

Information on tumor characteristics including histopathological information, hormone receptor-, HER2-and Ki-67 statuses was accessible for a subset of patients from the public laboratories of Tampere and Turku University Hospitals. To enable subtyping, the subcohort was limited to patients from these two University Hospital districts, which account for approximately 32 % of the Finnish population [21]. Patients with multiple tumors during follow-up and patients with missing hormone receptor-, HER2-, Ki-67, or disease extent information were excluded.

**Fig. 1:**
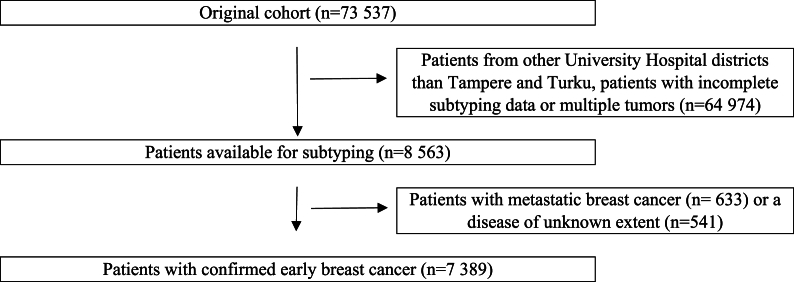
Flowchart describing the study population selection.

Each patient's follow-up period started from BC diagnosis and continued until death, emigration, or end of follow-up on December 31st, 2015, whichever occurred first. The flowchart of the study population is shown in Fig. 1. Information regarding deaths and the primary causes of deaths were obtained from the national death certificate registry maintained by Statistics Finland.

The study permission was granted by the Institute for 10.13039/100018696Health and Welfare (10.13039/501100014578THL) and the permission for data use was granted by the data registrars. Adhering to Finnish laws and regulations, the study did not require informed consent nor ethics committee review because of its retrospective nature as a national registry study.

### 2.2 Classification of tumors according to intrinsic subtypes

The classification of the tumors into different subtypes was undertaken following the clinicopathological surrogate definitions adapted from the 2013 St Gallen Consensus Conference and the ESMO guidelines [3,8,22]. We used the established cut-offs for ER-positivity (>10 %) and PR-positivity (≥20 %), while Ki-67 < 14 % was considered low and Ki-67 ≥ 14 % high according to the 2013 St Gallen Consensus Conference and the ESMO guidelines [3,8,22]. ER- and PR-positive, Ki-67 low and non-HER2 overexpressing tumors were deemed as Luminal A-like carcinomas. Luminal B-like (HER2-negative) carcinomas were either PR-negative or had Ki-67 ≥ 14 % while ER was positive and HER2 negative. Luminal B-like (HER2-positive) carcinomas were ER-positive, HER2 overexpressing tumors with any Ki-67 or PR expression. HER2-positive (non-luminal) carcinomas were ER- and PR-negative and HER2 overexpressing tumors with any Ki-67. Triple-negative carcinomas were ER-, PR- and HER2-negative with any Ki-67.

### 2.3 Mammographic screening coverage, attendance, and means of detection

To evaluate mammographic screening coverage, we evaluated whether the cancer was detected before (<49 years), during (49–70 years), or after (>70 years) the screening age. We then evaluated whether the individuals had attended the previous mammographic screening round or not (i.e., no screening-attendance within 2 years of the cancer diagnosis). The means of detection (i.e., whether the cancer was detected within the screening practice as a screen-detected cancer or in-between the screening rounds (i.e., interval cancer)) was recorded. Patients who did not attend any screening round were classified as patients eligible, but not attending screening.

### 2.4 Statistical analyses

Statistical analyses were performed with IBM SPSS for Windows, version 28.0 (IBM Corporation). We used the Cox proportional hazards regression model to evaluate the risk of death (hazard ratio [HR]) between different intrinsic subtypes and screening coverage, attendance, and means of detection. Survival was analyzed using the Kaplan-Meier curves and log-rank test. The p-value below 0.05 was predetermined to be statistically significant.

## 3 Results

The flowchart of the study cohort formation is shown in Fig. 1. The final study population consisted of 7389 patients with early BC. The median age at diagnosis was 60 (IQR: 52–70) (range 21–102) years. The median follow-up time was 6.5 (IQR: 3.1–9.7) years after the BC diagnosis. Altogether 1673 (22.6 %) died of any and 704 (42.1 %) of BC-related causes. Luminal B-like (HER2-negative) subtype was the most frequent subtype in this cohort with 3669 cases (49.7 %), while HER2-positive (non-luminal) was the least frequent subtype with 374 cases (5.1 %). The patient characteristics are shown in Table 1.

**Table 1:** Patient characteristics.

		Total	Luminal A-like	Luminal B-like (HER2-negative)	Luminal B-like (HER2-positive)	HER2-positive (non-luminal)	Triple-negative
**Patients**	n (% of total cases)	7389 (100)	2124 (28.7)	3669 (49.7)	563 (7.6)	374 (5.1)	659 (8.9)
**Age at diagnosis**	Median (IQR) [range]	60 (52–70) [21–102]	61 (54–69) [30–102]	61 (52–71) [21–98]	58 (50–67) [23–97]	60 (51–69) [27–92]	60 (50–71) [22–94]
**Histology**							
	Ductal (%)	5729 (77.5)	1527 (71.9)	2828 (77.1)	486 (86.3)	329 (88.0)	559 (84.8)
	Lobular (%)	1268 (17.2)	453 (21.3)	661 (18.0)	66 (11.7)	30 (8.0)	58 (8.8)
	Other (%)	392 (5.3)	144 (6.8)	180 (4.9)	11 (2.0)	15 (4.0)	42 (6.4)
**Deaths**							
	Total (%)	1673 (22.6)	405 (19.1)	859 (23.4)	134 (23.8)	86 (30.0)	189 (28.7)
	BC-related (%)	704 (9.5)	124 (5.8)	367 (10.0)	71 (12.6)	39 (10.4)	103 (15.6)
**Screening status**							
	BC before screening age (%)	1069 (14.5)	232 (10.9)	535 (14.6)	117 (20.8)	59 (15.8)	126 (19.1)
	BC in patients eligible, but not attending screening (%)	460 (6.2)	125 (5.9)	235 (6.4)	34 (6.0)	30 (8.0)	36 (5.5)
	Interval BC (%)	1829 (24.8)	546 (25.7)	876 (23.9)	145 (25.8)	110 (29.4)	152 (23.1)
	Screen-detected BC (%)	2244 (30.4)	736 (34.7)	1091 (29.7)	151 (26.8)	95 (25.4)	171 (25.9)
	BC after screening age (%)	1787 (24.2)	485 (22.8)	932 (25.4)	116 (20.6)	80 (21.4)	174 (26.4)

Abbreviations. BC = breast cancer, IQR = interquartile range.

### 3.1 Survival in different subtypes

**Fig. 2:**
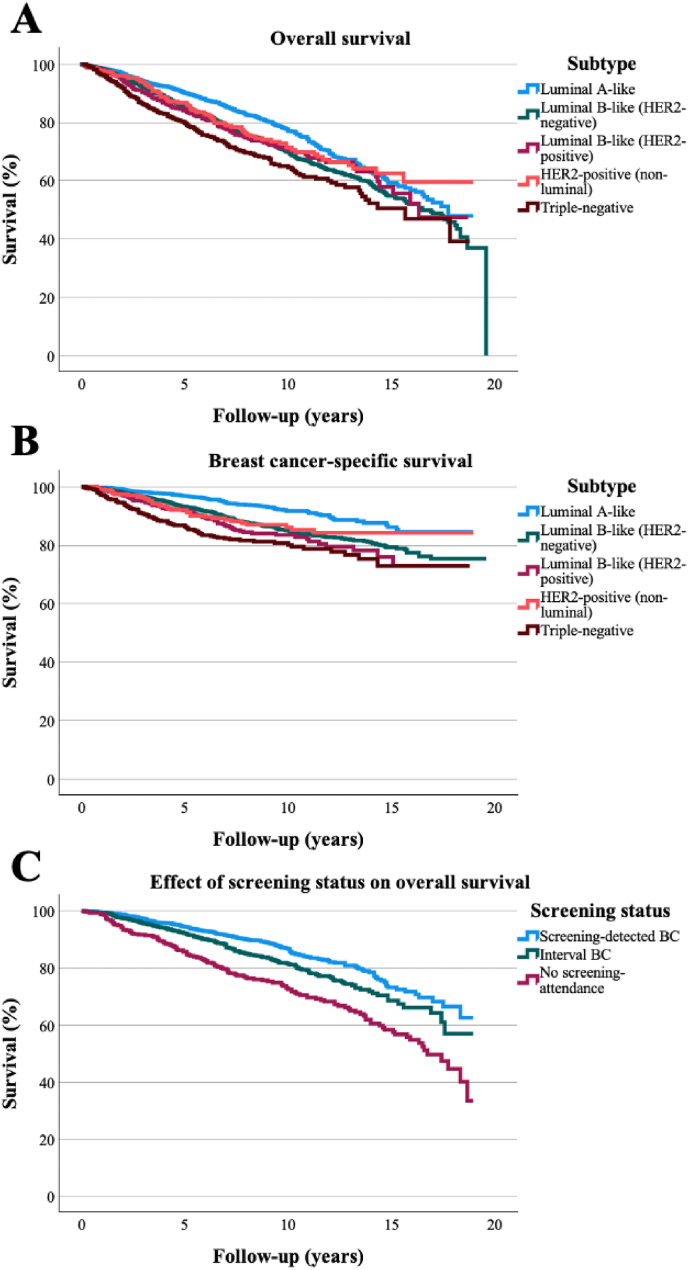
The survival differences between the intrinsic subtypes in the whole population (A: all-cause survival, B: breast cancer-specific survival) and between different screening statuses in screening-aged females (C). Abbreviations. BC = breast cancer. Patients with no screening-attendance were patients who were eligible for screening but had not attended any screening round.

The age at diagnosis -adjusted risks for all-cause and BC-related deaths are shown in Table 2. The intrinsic subtype influenced the risks of all-cause and BC-related deaths: compared to luminal A-like BC, those with triple-negative BC had the highest risks of all-cause (HR: 1.81, 95 % confidence interval (CI): 1.52–2.15) and BC-related (HR: 3.16, 95 % CI: 2.43–4.10) death. The Kaplan-Meier curve on the survival differences between the intrinsic subtypes is presented in Fig. 2.

**Table 2:** The risk for all-cause and breast cancer -related deaths according to the intrinsic subtypes adjusted for the age at the time of diagnosis.

	All-cause deaths n = 1673			Breast cancer -related deaths n = 704		
	HR	95.0 % CI	n	HR	95.0 % CI	n
Luminal A-like (n = 2124)	Ref		405	Ref		124
Luminal B-like (HER2-negative) (n = 3669)	1.33	1.18–1.50	859	1.89	1.54–2.32	367
Luminal B-like (HER2-positive) (n = 563)	1.66	1.36–2.02	134	2.51	1.87–3.36	71
HER2-positive (non-luminal) (n = 374)	1.36	1.07–1.71	86	1.85	1.29–2.66	39
Triple-negative (n = 659)	1.81	1.52–2.15	189	3.16	2.43–4.10	103

Abbreviations. HR = hazard ratio, CI = confidence interval.

### 3.2 Screening coverage, attendance and the means of cancer detection

The majority (n = 4533 (61.3 %)) of tumors were diagnosed in screening-aged females. Of them, nearly half (n = 2244 (49.5 %)) of all BCs were screen-detected whereas 460 (10.1 %) of cases were diagnosed in patients who did not attend screening and 1829 (40.3 %) of cases were interval cancers. The median age of patients with screen-detected BC, interval BC and patients with BC who were eligible, but not attending screening patients were 58 (IQR: 54–62), 61 (IQR:55–65) and 63 (IQR: 50–67), respectively. The distribution of intrinsic subtypes within different means of cancer detection including median, IQR and range of age at diagnosis is depicted in Supplement Table 1. Altogether 2856 (38.7 %) patients were diagnosed outside the national screening age (i.e., before the age of 49 years (n = 1069 (14.5 %)) or after the age of 70 years (n = 1787 (24.2 %)). The largest proportion of luminal A-like (34.7 %), luminal B-like (HER2-negative) (29.7 %) and luminal B-like (HER2-positive) (26.8 %) cancers were screen-detected. Most of HER2-positive (non-luminal) tumors were interval cancers (29.4 %), while most triple-negative tumors occurred after the screening age (26.4 %) (Table 1). In tumors detected before the screening age, HER2-positive (non-luminal) and triple-negative subtypes were overrepresented (Table 1). The association between screening coverage and survival is shown in Table 3. There was no statistically significant difference in survival between cases diagnosed in screening aged patients and patients diagnosed before the screening age. However, the risk of death was 5–7 times higher in patients diagnosed after the screening age compared to pre-screening-aged patients (Table 3).

**Table 3:** The association between screening coverage and the risk of death according to different intrinsic subtypes.

	BC before screening age		BC in screening age				BC after screening age			
	n	HR	n	HR	95 % CI	p	n	HR	95 % CI	p
All patients	1069	ref	4533	1.04	0.87–1.23	0.687	1787	5.5	4.66–6.49	<0.001
Luminal A-like	232	ref	1407	1.16	0.78–1.72	0.457	485	7.39	5.02–10.89	<0.001
Luminal B (HER2-negative)	535	ref	2202	0.94	0.75–1.18	0.594	932	5.01	4.01–6.26	<0.001
Luminal B (HER2-positive)	117	ref	330	1.11	0.65–1.88	0.703	116	6.73	3.99–11.35	<0.001
HER2-positive (non-luminal)	59	ref	235	1.35	0.63–2.90	0.436	80	5.99	2.80–12.85	<0.001
Triple-negative	126	ref	359	1.59	0.96–2.63	0.069	174	5.39	3.28–8.86	<0.001

Abbreviations. BC = breast cancer, HR = hazard ratio, CI = confidence interval.

In screening-aged females, the risk of death was higher in patients with interval cancers compared to screen-detected cases (HR 1.40, 95 % CI: 1.18–1.68) and even higher among those who did not attend screening (HR 2.17, 95 % CI: 1.75–2.68). This trend was similar in luminal HER2-negative tumors (luminal A and luminal B); however, interval BC or screening non-attendance did not translate to an increased risk of death among patients with HER2-positive (luminal B and non-luminal) and triple-negative BCs compared to screen-detected BCs. The association between the means of detection and screening-attendance are shown in Table 4 according to different intrinsic subtypes. The Kaplan-Meier curves depicting the survival differences between patients with different means of BC detection and screening attendance are shown in Fig. 2.

**Table 4:** The associations between the means of detection, screening attendance and the risk of death in screening-aged patients according to different intrinsic subtypes.

	Screen-detected BC		Interval BC				BC in patients not attending screening			
	n	HR	n	HR	95 % CI	p	n	HR	95 % CI	p
All screening-aged patients	2244	ref	1829	1.4	1.18–1.68	<0.001	460	2.17	1.75–2.68	<0.001
Luminal A-like	736	ref	546	1.52	1.04–2.21	0.029	125	2.52	1.63–3.89	<0.001
Luminal B (HER2-negative)	1091	ref	876	1.38	1.07–1.78	0.012	235	2.04	1.51–2.76	<0.001
Luminal B (HER2-positive)	151	ref	145	1.77	0.96–3.25	0.065	34	1.89	0.80–4.42	0.145
HER2-positive (non-luminal)	95	ref	110	1.63	0.79–3.37	0.184	30	2.04	0.83–4.99	0.118
Triple-negative	171	ref	152	0.85	0.52–1.39	0.520	36	1.83	1.00–3.35	0.051

Abbreviations. BC = breast cancer, HR = hazard ratio, CI = confidence interval.

## 4 Discussion

In our real-world retrospective data, the luminal B-like (HER2-negative) and luminal A-like subtypes were the most frequent subtypes. The intrinsic subtype influenced the risk of death and the risk of BC-related death: patients with luminal A-like tumors had the best prognosis, while patients with triple-negative tumors had the worst prognosis. In screening-aged females, patients with screen-detected tumors had the best survival, while patients with interval tumors and patients who did not attend screening had worse prognosis. This association on screening-related survival remained significant in luminal B-like (HER2-negative) and luminal A-like tumors but was not statistically significant in HER2-positive and triple-negative subtypes.

The distribution of intrinsic subtypes and the survival differences between subtypes were in-line with previous findings [10,12,13,15]. The survival difference between subtypes was even more prominent when considering only BC-related deaths (Table 2). Luminal A-like tumors had the best prognosis, while in long-term follow-up HER2-positive subtypes had better survival than the patients with luminal B (HER2-negative) subtype BCs. Trastuzumab-based adjuvant therapy has significantly improved survival in HER2-positive tumors [23,24], which likely explains the better survival of HER2-positive BC in longer follow-up.

In this large real-world population, the largest single group of patients in luminal A-like and both luminal B-like subtypes were screen-detected (Table 1). In the more aggressive subtypes, luminal B (HER2-positive), HER2-positive (non-luminal) and triple-negative, the proportion of patients diagnosed at screening or between screenings were interestingly quite similar. As expected, the proportion of luminal B (HER2-positive) and triple-negative subtypes were relatively higher in comparison to other subtypes among females younger than the screening age. The age at diagnosis was the lowest in the screen-detected BC group. Patients in the interval BC group and those eligible, but not attending screening were older. Mammographic screening results in earlier detection of BC [19,20]; this could explain the aforementioned age differences between different screening statuses, which were similar across patients with different intrinsic subtypes. In agreement with earlier studies, interval cancers possessed more adverse prognostic factors compared to screen-detected tumors although the differences were not so robust than previously reported (Table 2) [[25], [26], [27], [28]]. Interestingly, triple-negative tumors were more likely to be screen-detected rather than interval cancers, and most of them were diagnosed after the screening age (Table 1). To our knowledge, the distribution of cases diagnosed before, during, and after screening age has not previously been reported to this extent.

In agreement with earlier studies [[18], [19], [20]], screening status was significantly associated with survival of screening-aged BC patients (Table 3). Screen-detected tumors had a better prognosis than interval tumors, while the worst prognosis was seen in the patient group that did not attend screening. In addition to different distribution of intrinsic subtypes, interval cancers are known to harbor other adverse prognostic factors, such as larger tumor size, higher count of nodal metastases and higher histological grade [19]. We were able to show this same association between screening status and prognosis even within the largest intrinsic subtype groups (Table 4): patients with screen-detected luminal A-like and luminal B-like (HER2-negative) subtypes had better prognosis in comparison to patients with interval cancers and those not attending screening. A similar trend was observed also in the other subtypes but without statistical significance. We are not aware of earlier studies reporting the association between screening status and survival in different intrinsic subtypes.

The strength of our study was the use of a large real-world cohort consisting of patients with both subtype and screening data. In addition, we were able to include patients outside the screening age and patients who did not attend screening. To our knowledge, the previous data regarding the association of different subtypes and screening results is derived from relatively small subsets of patients in comparison to our population and it has not been possible to investigate the association between screening status and survival, especially in patients not attending screening, to this extent [[18], [19], [20],^28^]. The weakness of our study is that our dataset is retrospective. Most of the patients from the original cohort were excluded because of the lack of complete subtyping data due to inconsistent and imperfect registering. This exclusion can lead to a possibility of bias in our population, but this likely does not affect the conclusions on the associations between intrinsic subtypes, mammography screening, and prognosis. In addition, we had no reliable data on tumor size or lymph node involvement. Furthermore, data on disease relapses, treatments or comorbidities of the patients was not available. The median follow-up time was 6.5 (IQR 3.1–9.7) years, which is comparable with earlier studies, albeit we know that a smaller portion of relapses occur even after 10 years of follow-up, more frequently in luminal A-like subtype [12,13,15,29]. Future studies on the topic should aim at reporting results with preferably longer follow-up time, and disclose important background information, including information on tumor size, nodal involvement, disease relapses, treatments, and comorbidities.

In conclusion, our findings in this large real-world dataset agreed with survival differences between patients with different intrinsic subtypes reported earlier. We showed lower survival rates in patients with luminal A-like and luminal B-like (HER2-negative) interval cancers and those not attending screening compared to patients with screen-detected cancers.

## CRediT authorship contribution statement

**Santeri Palmi:** Writing – original draft, Visualization, Methodology, Investigation, Conceptualization. **Teemu J Murtola:** Writing – review & editing, Validation, Methodology, Investigation, Formal analysis, Data curation, Conceptualization. **Mika Murto:** Writing – review & editing, Methodology, Conceptualization. **Heini Huhtala:** Writing – review & editing, Visualization, Validation, Methodology. **Otso Arponen:** Writing – review & editing, Visualization, Validation, Supervision, Methodology, Investigation, Formal analysis, Data curation, Conceptualization. **Arja Jukkola:** Writing – review & editing, Supervision, Methodology, Investigation, Funding acquisition, Conceptualization.

## Ethical approval

The study permission was granted by the Institute for Health and Welfare (THL). Adhering to Finnish laws and regulations, the study did not require informed consent nor ethics committee review because of its retrospective nature as a national registry study.

## Data availability statement

The data are not publicly available due to restrictions aiming at ensuring patient confidentiality. Relevant data are presented in the manuscript.

## Conflict of interest

None.
