# Supplementary material for: The association between intrinsic breast cancer subtypes, mammography screening and prognosis: a large population-based real world cohort study
Source: Breast. 2025 May 23;82:104507. doi: 10.1016/j.breast.2025.104507 (PMC12153371; doi:10.1016/j.breast.2025.104507)
Supplement: Multimedia component 1 [file mmc1.docx]

|  | **Total** | **Luminal A-like** | **Luminal B-like (HER2-negative)** | **Luminal B-like (HER2-positive)** | **HER2-positive (non-luminal)** | **Triple-negative** |
| --- | --- | --- | --- | --- | --- | --- |
| **BC before screening age (%)** | 1 069 (14.5) | 232 (10.9) | 535 (14.6) | 117 (20.8) | 59 (15.8) | 126 (19.1) |
| Median age (IQR)  [age-range] | 44 (40-47)  [21-48] | 45 (41-47)  [30-48] | 44 (40-47)  [21-48] | 43 (39-46)  [23-48] | 42 (39-46)  [27-48] | 42 (39-45)  [22-48] |
| **BC in patients eligible, but not attending screening (%)** | 460 (6.2) | 125 (5.9) | 235 (6.4) | 34 (6.0) | 30 (8.0) | 36 (5.5) |
| Median age (IQR)  [age-range] | 63 (50-67)  [49-70] | 64 (55-68)  [49-70] | 63 (50-67)  [49-70] | 60 (50-67)  [49-70] | 64 (57-68)  [49-70] | 61 (49-67)  [49-70] |
| **Interval BC (%)** | 1 829 (24.8) | 546 (25.7) | 876 (23.9) | 145 (25.8) | 110 (29.4) | 152 (23.1) |
| Median age (IQR)  [age-range] | 61 (55-65)  [49-70] | 61 (56-65)  [49-70] | 61 (55-65)  [49-70] | 60 (54-64)  [49-70] | 61 (56-66)  [49-70] | 60 (54-64)  [49-70] |
| **Screen-detected BC (%)** | 2 244 (30.4) | 736 (34.7) | 1 091 (29.7) | 151 (26.8) | 95 (25.4) | 171 (25.9) |
| Median age (IQR)  [age-range] | 58 (54-62)  [49-70] | 58 (54-61)  [49-70] | 58 (54-62)  [49-70] | 56 (54-60)  [50-70] | 57 (52-62)  [49-70] | 58 (54-62)  [49-70] |
| **BC after screening age (%)** | 1 787 (24.2) | 485 (22.8) | 932 (25.4) | 116 (20.6) | 80 (21.4) | 174 (26.4) |
| Median age (IQR)  [age-range] | 78 (74-83)  [71-102] | 78 (74-82)  [71-102] | 78 (74-83)  [71-98] | 78 (75-83)  [71-97] | 76 (74-80)  [71-92] | 78 (74-82)  [71-94] |
| Abbreviations. BC=breast cancer, IQR=interquartile range | | | | | | |
| Supplementary Table 1. Median ages of patients with breast cancer according to intrinsic biological subtypes and means of cancer detection. | | | | | | |
